# Genomic epidemiology of extended-spectrum beta-lactamase-producing Escherichia coli from humans and a river in Aotearoa New Zealand

**DOI:** 10.1099/mgen.0.001341

**Published:** 2025-01-10

**Authors:** Holly A. Gray, Patrick J. Biggs, Anne C. Midwinter, Lynn E. Rogers, Ahmed Fayaz, Rukhshana N. Akhter, Sara A. Burgess

**Affiliations:** 1 ^*m*^EpiLab, School of Veterinary Science, Massey University, Palmerston North, New Zealand; 2 School of Food Technology and Natural Sciences, Massey University, Palmerston North, New Zealand; 3 New Zealand Food Safety Science and Research Centre, Massey University, Palmerston North, New Zealand

**Keywords:** antimicrobial resistance, *E. coli*, ESBL, freshwater, urinary tract infection

## Abstract

In Aotearoa New Zealand, urinary tract infections in humans are commonly caused by extended-spectrum beta-lactamase (ESBL)-producing *Escherichia coli*. This group of antimicrobial-resistant bacteria are often multidrug resistant. However, there is limited information on ESBL-producing *E. coli* found in the environment and their link with human clinical isolates. In this study, we examined the genetic relationship between environmental and human clinical ESBL-producing *E. coli* and isolates collected in parallel within the same area over 14 months. Environmental samples were collected from treated effluent, stormwater and multiple locations along an Aotearoa New Zealand river. Treated effluent, stormwater and river water sourced downstream of the treated effluent outlet were the main samples that were positive for ESBL-producing *E. coli* (7/14 samples, 50.0%; 3/6 samples, 50%; and 15/28 samples, 54%, respectively). Whole-genome sequence comparison was carried out on 307 human clinical and 45 environmental ESBL-producing *E. coli* isolates. Sequence type 131 was dominant for both clinical (147/307, 47.9%) and environmental isolates (11/45, 24.4%). Only one ESBL gene was detected in each isolate. Among the clinical isolates, the most prevalent ESBL genes were *bla*_CTX-M-27_ (134/307, 43.6%) and *bla*_CTX-M-15_ (134/307, 43.6%). Among the environmental isolates, *bla*_CTX-M-15_ (28/45, 62.2%) was the most prevalent gene. A core SNP analysis of these isolates suggested that some strains were shared between humans and the local river. These results highlight the importance of understanding different transmission pathways for the spread of ESBL-producing *E. coli*.

**Impact Statement**

Extended-spectrum beta-lactamase (ESBL)-producing *Escherichia coli* frequently cause urinary tract infections that exhibit multidrug resistance. Surveillance studies have identified the predominant strains and resistance genes associated with urinary tract infections. However, there is limited information on the extent of spread beyond the patient. We describe the genetic relatedness of ESBL-producing environmental and clinical *E. coli* isolated during the same temporospatial period in Aotearoa New Zealand. Comparative genomic analyses of these bacteria provide evidence of clonal spread between humans and the environment, highlighting the need to integrate environmental surveillance into antimicrobial resistance monitoring.

## Data Summary

All Illumina sequence reads for this study have been deposited in GenBank under BioProject PRJNA1032159, except for strain SB0283h1, whose data can be found under BioProject PRJNA715472. The sequence read accessions for each genome are provided in the supplementary material. The code used for the genomic and statistical analyses is available from the GitHub repository https://github.com/sburgess1/Manawat-_ESBL. The authors confirm all supporting data and protocols have been provided within the article or through supplementary data files.

## Introduction

*Escherichia coli* are Gram-negative bacteria that form a natural part of the mammalian intestinal tract microbiota. They can be opportunistic pathogens and can cause a range of infections in humans, such as pneumonia, bacteraemia, meningitis and urinary tract infections [1,3]. The spread of antimicrobial resistance in pathogenic strains of *E. coli* is of importance because infections caused by these strains are often harder to treat, resulting in increased severity and duration of infection [4,5]. One important group of antimicrobial-resistant *E. coli* is the extended-spectrum beta-lactamase (ESBL)-producing *E. coli*. In Aotearoa New Zealand, *E. coli* was associated with 84.6% of ESBL-producing *Enterobacterales*-related infections in 2019 [6].

The ESBL enzymes confer resistance to an extended range of beta-lactam antibiotics, including the first-, third- and some fourth-generation cephalosporins but not the cephamycins or carbapenems [7,8]. CTX-M is the predominant enzyme type associated with human clinical ESBL-producing *E. coli* [9,11]. There are more than 250 *bla*_CTX-M_ gene variants, with *bla*_CTX-M-15_ being the most common among human clinical *E. coli* isolates [12,15]. The genes encoding ESBLs are often associated with other antimicrobial resistance genes (ARGs), resulting in a multidrug-resistant (MDR) strain [10,11, 16]. MDR pathogenic strains of *Enterobacteriaceae* are of particular concern to the human health sector resulting in ESBL-producing *Enterobacteriaceae* being identified as ‘critical’ on the World Health Organization’s ‘Priority Pathogens List’ [17].

The main route for community dissemination of antimicrobial-resistant bacteria involves person-to-person transmission [18,19]. However, there are alternative transmission pathways, such as contact with companion and livestock animals, consumption of food products, or indirect exposure through contaminated recreational water [20,23]. The natural environment and particularly recreational water have been identified as important vectors in the transmission of antimicrobial resistant bacteria [24,25]. Recreational water use has rarely been found to be a risk factor for ESBL infection and the carriage of ESBL-producing *E. coli* in recreational water users has not been found to be higher compared to non-recreational water users [26,29]. However, freshwater has been identified as an environmental source of ESBL-producing *E. coli* [30,31]. There are few studies that have compared ESBL-producing *E. coli* from human clinical samples and freshwater samples [32,34]. The aim of this study was to compare the genetic relatedness of clinical and environmental ESBL-producing *E. coli*. Whole genome sequence analysis was carried out for isolates collected over the same time period and geographical region.

## Methods

### Sample collection and processing

**Fig. 1.**
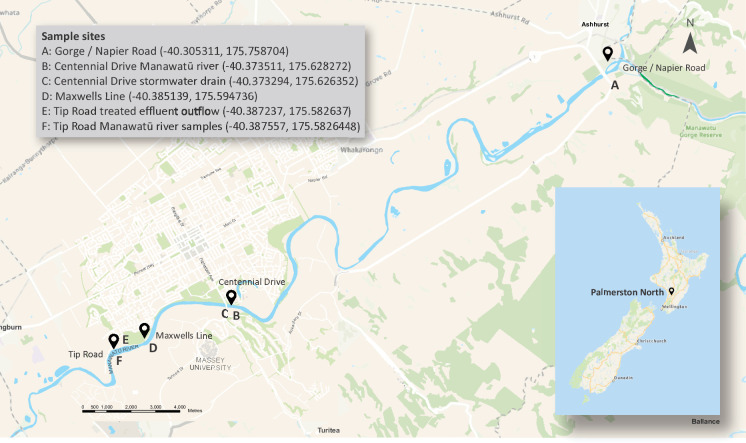
Sampling sites A to F along the Manawatū River, located in Palmerston, North Aotearoa New Zealand. River flow is the direction of site A to F. Image produced using CorelDRAW 2019 (Corel Corporation). Base map obtained from Google Maps (2023), available at https://www.google.com/maps/@-40.3521071,175.6424104,12.87z?entry=ttu (accessed on 16 August 2023).

Environmental samples were collected monthly from the Manawatū River (sites A, B, D and F), stormwater (site C) and treated effluent (site E) over 14 months: August 2019 to March 2020 and July 2020 to January 2021 (excluding October 2019). Two water samples and one sediment sample were collected from locations A (upstream of the city of Palmerston North) and B, D and F (downstream) along the Manawatū River (Fig. 1). In total, 28 water samples were collected from sites A, B and F; 22 water samples were collected from site D. Treated effluent was sampled from a drain after exiting the wastewater treatment plant before entering the river. The Palmerston North wastewater treatment process involves screening, primary and secondary sedimentation and ultraviolet treatment. It then passes through a wetland before entering the Manawatū River. In total, 14 effluent samples were collected from site E. A stormwater sample was collected six times from the Centennial Drive site (site C) when the stormwater flowed from the drain. The Palmerston North Hospital is within the Manawatū River basin (~2.8 km from the river itself), and the wastewater from the hospital is treated at the Palmerston North Wastewater Treatment Plant.

Water samples prepared as previously described [35] and sediment (1 g) were enriched in 10 ml of buffered peptone water (BPW) (BD Difco™, Becton Dickinson) as well as 10 ml of *E. coli* broth (ECB) (Oxoid Ltd) and incubated overnight at 35 °C. Additionally, 1 ml of treated effluent was diluted in 9 ml of BPW or ECB and incubated overnight at 35 °C. Two millilitres of each enrichment was centrifuged for 5 min at 6000 ***g***, and the pellet was resuspended in 1 ml of in-house prepared nutrient broth no. 2 (Oxoid Ltd) containing 15% (v/v) glycerol and was then stored at −80 °C. Each sample was allocated a unique identifying number with the prefix ‘SB’ followed by the allocated sample number. The water filter, sediment and effluent enriched in BPW and ECB were plated onto two selective MacConkey agar plates (containing 1 µg ml^−1^ of cefotaxime sodium or ceftazidime) and CHROMagar™ ESBL (Fort Richard Laboratories) as previously described [36]. Additionally, 1 ml of treated effluent was plated onto CHROMagar™ ESBL. The selective agar plates were incubated at 35 °C for 16 to 24 h, and up to two presumptive *E. coli* colonies from each plate were purified on Columbia horse blood agar (Fort Richard Laboratories), incubated at 35 °C for 16 to 24 h and identified using MALDI-TOF Biotyper® (Bruker Daltonics) [36,37]. Presumptive ESBL-producing *Enterobacteriaceae* clinical strains, which originated from urine, blood or wound swab cultures, were isolated by the MedLab Central Laboratory (Palmerston North, Aotearoa New Zealand), from August 2019 to March 2020 and June 2020 to January 2021. MedLab Central processes samples from the Manawatū, Hawke’s Bay, Gisborne and Whanganui regions. These isolates were collected weekly and were also purified on Columbia horse blood agar, identiﬁed using MALDI-TOF mass spectrometry, and allocated a unique identifying number with the prefix ‘EH’. A total of 415 isolates were collected, of which 359 isolates were *E. coli*.

### Antimicrobial susceptibility testing

Antimicrobial susceptibility tests and confirmation of an ESBL-producing phenotype were carried out using Kirby–Bauer disc diffusion assays following Clinical and Laboratory Standards Institute guidelines [38]. Antimicrobial susceptibility tests of the environmental *E. coli* isolates were carried out using a panel of ten antibiotics (MAST Group, Table S1, available in the online Supplementary Material). An MDR isolate was defined as being resistant to three or more classes of antibiotics. ESBL confirmation was carried out on the clinical *E. coli* as well as those environmental *E. coli* that were non-susceptible to cefotaxime or ceftazidime using the double-disc comparison assay (D62C cefotaxime and D64C ceftazidime ESBL disc tests; Mast Group).

### DNA extractions and whole-genome sequencing

ESBL-producing *E. coli* isolates were grown in Luria–Bertani broth (made with deionized water, NaCl [Scharlau], yeast extract [Becton Dickinson Difco™] and tryptone [Becton Dickinson Difco™]) overnight at 35 °C, and genomic DNA was extracted using the Wizard® Genomic DNA purification kit (Promega) as previously described [35]. Libraries were generated using the Nextera XT DNA library Preparation Kit (Illumina Inc.) and submitted to the Massey Genome Service (Massey University) for quality checks and sequencing. Whole-genome sequencing was performed by Novogene (Singapore) using Illumina HiSeq™ X sequencing (2×150 bp paired-end reads). FastQC (v.0.11.9) was used for the quality assessment of the raw reads [39].

### Genome assemblies and analyses

The raw reads were processed using Nullarbor 2 (v.2.0.20191013), using the default parameters, and the strain *E. coli* EC958 was used as the reference (GenBank accession HG941718.1) [40,41]. Using this pipeline, the adapters were removed using Trimmomatic (v.0.39), the reads were assembled using SKESA (v.2.4.0), and the assemblies were annotated using Prokka (v.1.14.6) [42,44]. Quast (v.5.0.2) and CheckM (v.1.2.3) were used for genome evaluations [45,46]. Assemblies were quality assessed by examining the overall length (expected to be 5 Mb), the GC percentage (expected to be 50.0–51.0%), the number of coding sequences (expected to be 4000–5000), contamination (less than 5%) and the number of contigs (expected to be less than 500). No assemblies were discarded. Additionally, multilocus sequence typing (MLST) was carried out using mlst (v.2.19.0), phylogrouping was carried out using EzClermont (v.0.7.0) [47], and resistance and virulence genes were identified using ABRicate (v.1.0.1) with the National Center for Biotechnology Information AMRFinderPlus database (v.2021-03-27) [48] and the virulence factor database (VFDB, v.2021-03-27) [49], respectively. Twenty-one virulence genes were used to define whether the isolates belonged to a particular pathotype (Table S2) [50,53]. PlasmidFinder (v.2.1.6) and pMLST (v.2.0.3, using the python script version downloadable from https://bioconda.github.io/recipes/pmlst/README.html) were used for identifying and typing plasmids [54]. PointFinder (v.3.0) was used to detect point mutations associated with antimicrobial resistance in the sequences of the ST131 isolates [55]. The *fimH* type of all ST131 isolates was determined using FimTyper (v.1.0.1) with default parameters [56]. ST131 were divided into clades A, B and C based on their *fimH* type, mutations in the *gyrA* and *parC* genes as well as their position within a core SNP phylogeny. Clade C ST131 genomes were further divided into C1 and C2 based on two specific SNPs within the *sbmA* and *ybbW* genes [57].

ChewBBACA (v.3.1.2) was used to generate core-genome MLST (cgMLST) allele profiles for all the genomes as well as the environmental genomes only, using a 95% threshold [58]. The cgMLST profiles were used to generate distance matrices using cgMLST-dists (v.0.4.0, https://github.com/tseemann/cgmlst-dists) [58], which were presented as neighbour-joining trees using the R package ape (v.5.7–1) [59].

A core SNP analysis of the ST131, ST69 and ST1193 isolates was carried out using Snippy-multi (v.4.6.0), employing the internal references EH0395a (ST131), EH0143a (ST69) and EH0294a (ST1193), to determine the number of SNPs between isolates [60]. We defined a possible transmission event as two *E. coli* strains isolated within the same month, with a difference of less than 10 SNPs between them. Gubbins (v.2.3.1) was applied to all Snippy analyses, using the default settings. FastTree (v.2.1.11) was used to construct maximum likelihood phylogenetic trees from the core SNP alignment using a general time-reversible substitution model with the default settings. The phylogenetic trees were uploaded to the Interactive Tree Of Life (iTOL) (v.6.5.7) for annotation and visualization [59]. To classify the ST131 isolates into clades, sequence reads of known ST131 clades A, B and C were downloaded from the European Nucleotide Archive: strains MER-56 (SRR5936479), MER-53 (SRR5936492) and MER-25 (SRR5936501), respectively [14].

### ESBL gene analysis

Contigs containing the *bla*_CTX-M_ gene, with a minimum length of 10 kbp, were extracted using a custom Python script (v.3.8.1) and annotated using Prokka (v.1.14.6). The General Feature Format Prokka-generated files were used as the input for a pangenome analysis using Panaroo (v.1.3.0), which was run in sensitive mode, using the aligner MAFFT, and a core threshold of 0.95 [61,62]. The gene presence–absence output from Panaroo was uploaded into Panini (https://panini.cgps.group/accessed on 27 October 2023), and the resulting csv and dot files were uploaded to Microreact (https://microreact.org/accessed on 27 October 2023) [63,64].

### Graphical data display

Phenotypic results were stored in a Microsoft Access database (Microsoft 365, v16.0.1) and analysed using the software R (v.4.1.2, R computing group). The code used is available from the GitHub repository: https://github.com/sburgess1/Manawat-_ESBL. R packages used included ggplot2 (v.3.3.5), lubridate (v.1.8.0), ComplexUpset (v.1.3.1), ggalluvial (v.0.12.3) and easyalluvial (v.0.3.0).

### Statistical analyses

Statistical analyses were performed using Rstudio (v.2023.06.1). Wilson score confidence intervals (CIs) were generated using the prop.test function. The Fisher’s exact test using the fisher.test function was used to test the null hypothesis that there was no difference in the prevalence of ESBL-producing *E. coli* between the sample site downstream of the wastewater treatment plant (site F) and the other river sample sites (A, B and D).

## Results

### The detection of ESBL-producing and MDR *E. coli* in freshwater, stormwater and treated effluent

Antimicrobial-resistant *E. coli*, including ESBL-producing and MDR strains, were detected in water and sediment samples from the Manawatū River over a 14-month period, with a significantly higher prevalence observed downstream of the wastewater treatment plant. *E. coli* was isolated from 146 (146/174, 83.9%) environmental samples, with 28/146 (60.9%) of samples with *E. coli* detected containing ESBL-positive *E. coli*. ESBL-producing *E. coli* were isolated throughout every season, during periods of both low and high rainfall (Fig. S1, Table S3). MDR and ESBL-producing *E. coli* were detected from three of the four river sample sites (Table 1), with sample site F (downstream of the treated effluent outlet) having a significantly higher proportion of both ESBL-producing (15/28, 53.6%, 95% CI: 34.2–72.0%, *P*<0.001) and MDR *E. coli* (11/28, 39.3%, 95% CI: 22.1–59.3%, *P*<0.001) than the other three sites. To evaluate incoming environmental sources of antimicrobial-resistant *E. coli*, samples were also collected from a stormwater drain (site C) and the treated effluent outlet (site E) (Fig. 1). A similar proportion (*P*>0.05) of ESBL-producing *E. coli* and MDR *E. coli* was observed for both sites when compared with sample site F.

**Table 1.** The number of samples positive for ESBL-producing, antimicrobial and MDR *E. coli*

	A			B			C†	D			E	F		
**Sample type**	Sediment	Water	Total	Sediment	Water	Total	Storm water	Sediment	Water	Total	Treated effluent	Sediment	Water	Total
**ESBL**	0/12 (0.0%)	1/28 (3.6%)	1/40 (2.5%)	0/13 (0.0%)	1/28 (3.6%)	1/41 (2.4%)	**3/6** (50.0%)	0/11 (0.0%)	0/22 (0.0%)	0/33 (0.0%)	**7/14** (50.0%)	1/12 (8.3%)	**15/28** (53.6%)	16/40 (40.0%)
Antimicrobial resistant*	2/12 (16.7%)	2/28 (7.1%)	2/40 (5.0%)	2/13 (15.4%)	2/28 (7.1%)	4/41 (9.8%)	**4/6** (66.7%)	0/11 (0.0%)	0/22 (0.0%)	0/33 (0.0%)	**7/14** (50.0%)	2/12 (16.7%)	**17/28** (60.7%)	19/40 (47.5%)
**Multidrug resistant**	0/12 (0.0%)	1/28 (3.6%)	1/40 (2.5%)	0/13 (0.0%)	1/28 (3.6%)	1/41 (2.4%)	2/6 (33.3%)	0/11 (0.0%)	0/22 (0.0%)	0/33 (0.0%)	6/14 (42.9%)	1/12 (8.3%)	11/28 (39.3%)	12/40 (30.0%)

* Resistant to at least one of the ten antibiotics used to screen the samples, which includes any MDR isolates.

† Those sample level prevalences that are ≥50% have been bolded.

**Fig. 2.**
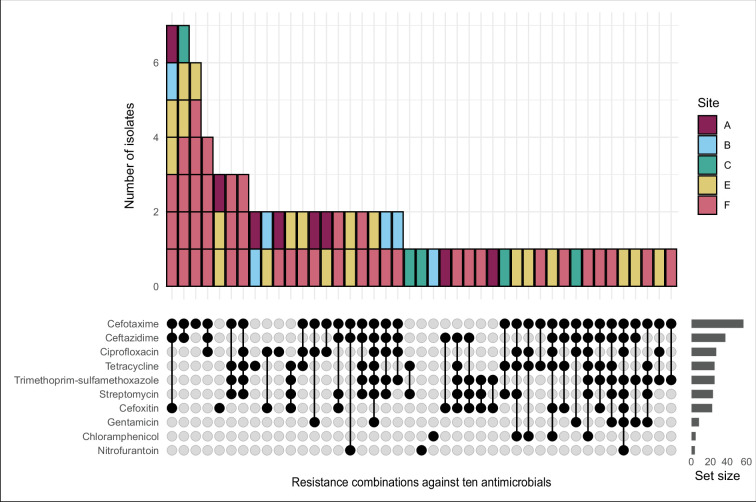
Antimicrobial resistance profiles of ESBL-producing *E. coli* against ten antibiotics from sample sites A to F along the Manawatū River. No antimicrobial-resistant isolates were isolated from sample site D.

The environmental *E. coli* isolated from the six sample sites were screened for resistance to ten antibiotics (Fig. 2). The most observed resistance phenotypes were to cefotaxime, ceftazidime and cefoxitin as well as cefotaxime and ceftazidime. Resistance to trimethoprim-sulfamethoxazole, which is commonly used for the treatment of urinary tract infections (UTIs) [65], was observed in combination with resistance to one or more other antimicrobials. A multidrug resistance phenotype was observed in 35/82 (42.7 %) isolates.

### Genetic diversity of ESBL-producing environmental *E. coli*

**Fig. 3.**
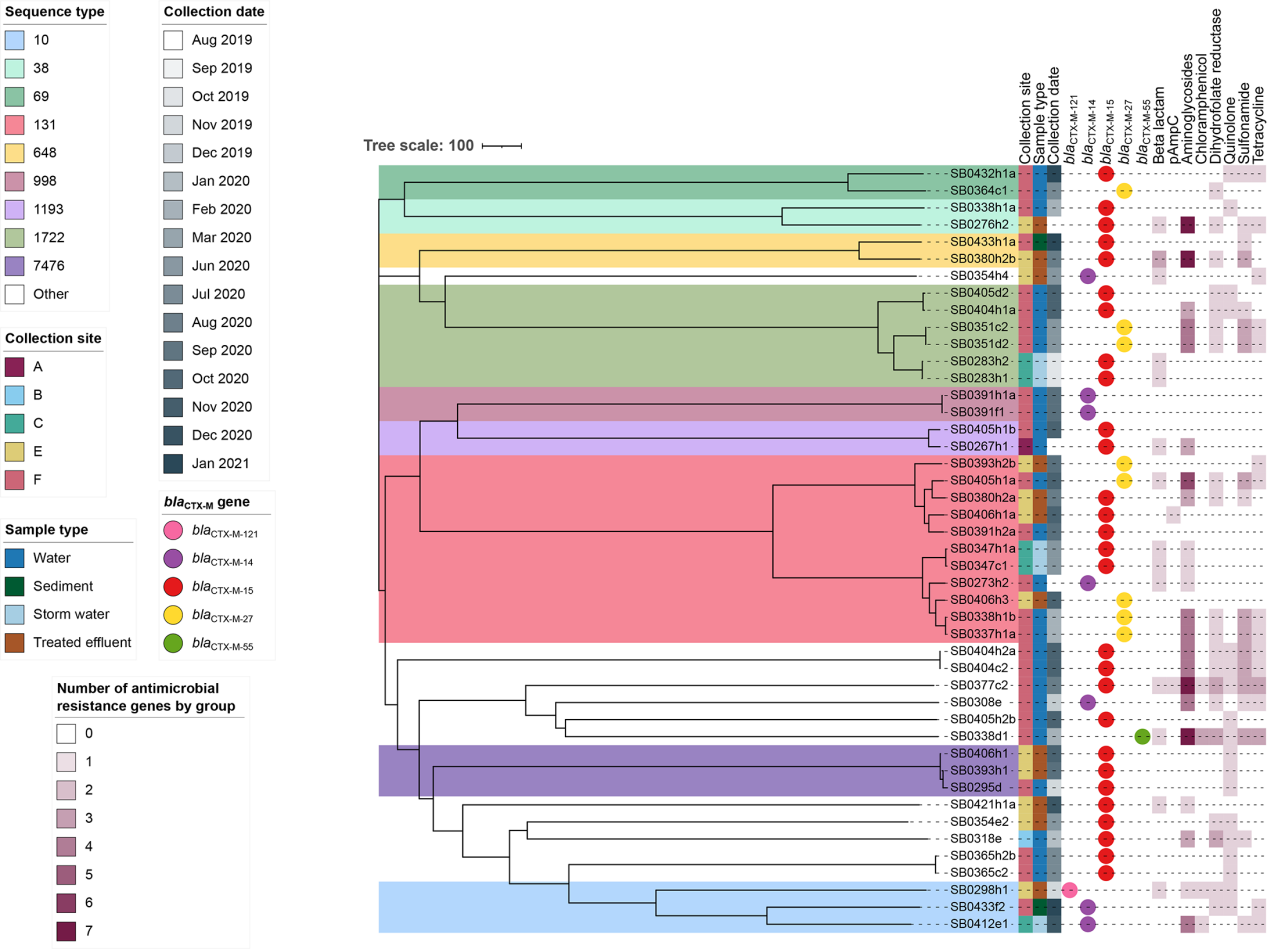
Core-genome MLST neighbour-joining tree of 45 environmental *E. coli* isolated from river water, sediment, stormwater and treated effluent. The tree was generated using 2918 shared alleles identified using chewBBACA and visualized in iTOL. The scale bar represents the number of allele differences. The coloured boxes represent the sequence type (those sequence types with 1–2 genomes were not coloured). From left to right, the coloured strips represent the collection site, the sample type and the collection date. The circles represent the *bla*_CTX-M_ gene variant, and the heatmap represents the presence/absence of genes conferring resistance to other groups of antibiotics. The ARG group ‘Beta lactam’ includes the genes *bla*_TEM-1_, *bla*_OXA-1_ and *bla*_OXA-10_, and the group ‘pAmpC’ (plasmid-mediated AmpC) includes the genes *bla*_CMY-2_ and *bla*_CMY-138_.

Whole-genome sequencing and a cgMLST analysis (using 2918 shared alleles, Fig. 3) of 45 environmental (26/45 from river water, 2/45 from sediment, 5/45 from stormwater, 12/45 from effluent, Table 2) ESBL-producing *E. coli* revealed a diverse population with 19 different sequence types (STs) and 7 different *bla*_CTX-M_ gene variants. ST131 was the predominant ST (11/45, 24.4%) followed by ST1722 (6/45, 13.3%), ST10 (3/45, 6.7%) and ST7476 (3/45, 6.7%). The predominant ESBL-coding gene was *bla*_CTX-M-15_ (28/45, 62.2%) followed by *bla*_CTX-M-27_ (8/45, 17.8%) and *bla*_CTX-M-14_ (7/45, 15.6%). Twenty-one of the isolates had a multidrug resistance genotype. The number of ARGs ranged from 1 to 19, covering 10 antibiotic classes: beta-lactams, aminoglycosides, rifamycins, tetracyclines, sulphonamides, trimethoprim, amphenicols, macrolides, phosphonic acids and lincosamides. However, the resistance genotype was not always concordant with the resistance phenotype. For example, trimethoprim-/sulfamethoxazole-resistant *E. coli* isolates did not always have a *dfr* and/or a *sul* gene present.

**Table 2.** The number of ESBL-producing *E. coli* isolates sequenced per environmental sample type

A		B		C	D		E	F	
Sediment	Water	Sediment	Water	Storm water	Sediment	Water	Treated effluent	Sediment	Water
0	1	0	1	5	0	0	12	2	24

### The genetic relationship of ESBL-producing clinical and environmental *E. coli*

**Fig. 4.**
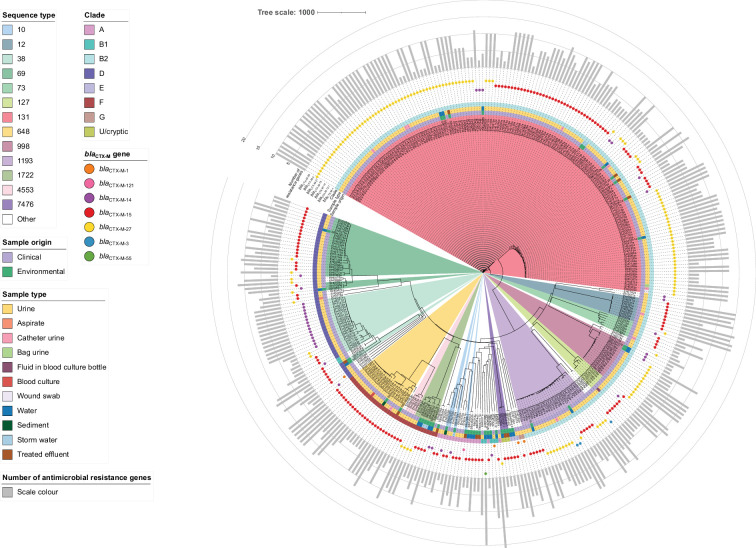
Core-genome MLST neighbour-joining tree of 352 clinical and environmental *E. coli*. The tree was generated using 3053 shared alleles identified using chewBBACA and visualized in iTOL. The scale bar represents the number of allele differences. The inner coloured circle represents the sequence type. Those sequence types with 1–2 genomes were not coloured. The coloured rings (from inner to outer) represent the sample origin, the sample type and the clade. The circles represent the *bla*_CTX-M_ gene variant, and the outer bar graph represents the number of ARGs detected.

To determine whether there was a spread of ESBL-producing *E. coli* between humans and waterways, a comparative genomic analysis was carried out, encompassing 307 human clinical ESBL-producing *E. coli* and the 45 environmental isolates (Table S4). Over the 14 months of sampling, there appeared to be no seasonal trend, with ST131 (158/352, 44.9%) being the dominant ST (Fig. S2). A cgMLST analysis (using 2918 shared alleles) demonstrated that the environmental isolates were dispersed throughout the phylogeny (Fig. 4). There were 7 river-only isolate STs (ST156, ST219, ST442, ST542, ST1324, ST1584 and ST2079), 2 effluent-only STs (ST540 and ST635) and 28 human-only STs. The predominant STs across all the isolates were ST131 (158/352, 44.9%), ST1193 (26/352, 7.4%), ST69 (25/352, 7.1%), ST38 (23/352, 6.5%), ST648 (21/352, 6.0%) and ST998 (15/352, 4.3%). Three STs, ST38, ST131 and ST648, were shared across the three sample types (clinical, effluent and river). Three STs were shared across the clinical and river isolates (ST1193, ST69 and ST998). The predominant ESBL-coding genes from the clinical isolates were *bla*_CTX-M-15_ (134/307, 43.6%) and *bla*_CTX-M-27_ (134/307, 43.6%) followed by *bla*_CTX-M-14_ (31/307, 10.1%). The number of ARGs ranged from 1 to 20, covering resistance to ten antimicrobial classes. For the three main STs, the predominant ESBL-coding gene was *bla*_CTX-M-27_ for ST131, *bla*_CTX-M-15_ for ST69 and *bla*_CTX-M-27_ for ST1193.

The majority of ST131 isolates fell within clade C (103/158, 65.2%) followed by clade A (51/158, 32.3%), with 4/158 (2.5%) belonging to clade B, all being clinical isolates (Table S5). The *bla*_CTX-M-27_ was the dominant ESBL-coding gene for both the clade A (38/51, 74.5%) and clade C (61/103, 59.2%) isolates. All of the clade C ST131 isolates harboured five point mutations: two in *gyrA*, two in *parC* and one in *parE*; whereas the majority of clade A (44/51, 86.3%) isolates carried two mutations, one each in *gyrA* and *parE*. The ST131 isolates harboured genes typical of ExPEC and UPEC (Table S6), which included the key genes *papA* (1/158, 0.6%), *papC* (77/158, 48.7%), *afaC* (21/158, 13.3%), *kpsM* (146/158, 92.4%), *iutA* (1/158, 0.6%), *chuA* (158/158, 100%), *fyuA* (158/158, 100%) and *sat* (132/158, 83.5%).

**Fig. 5.**
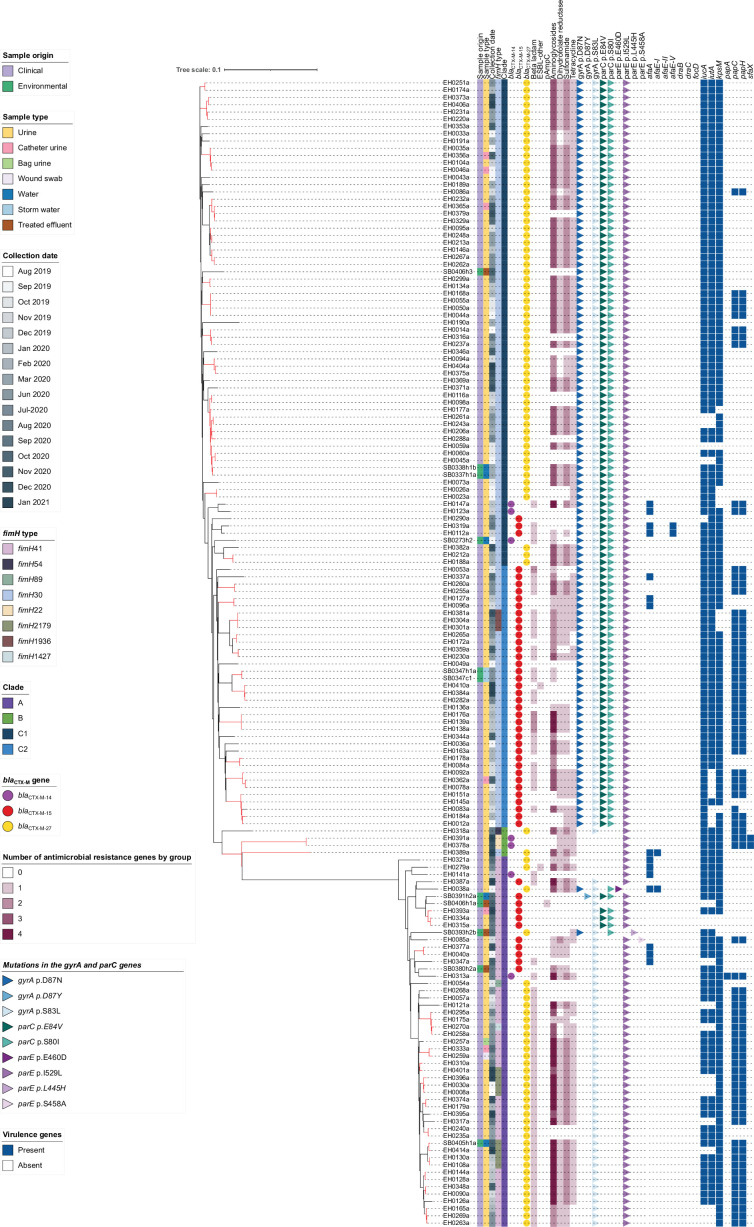
Core SNP phylogeny of clinical and environmental ST131 isolates. Maximum likelihood phylogenetic tree of 159 ST131 *E. coli* produced using 2370 SNPs with EH0395a used as the reference genome. The tree was constructed with FastTree using a maximum likelihood GTR model and visualized in iTOL. Branches coloured in red have a difference of fewer than 10 SNPs between genomes. The scale bar represents the proportion of nucleotide differences in the core SNP alignment used to calculate the divergence of the isolates. From left to right, the coloured strips represent the sample origin, the sample type, the collection date, *fimH* type and the clade. The circles represent the *bla*_CTX-M_ gene variant; the heatmap represents the presence/absence of genes conferring resistance to other groups of antibiotics; the triangles represent point mutations in the *gyrA*, *parC* and *parE* genes; and the blue squares represent the presence/absence of ExPEC associated virulence genes (*afaE-I*, *afaE-II*, *afaE-V*, *draB*, *draC*, *focD*, *iucA*, *iutA*, *kpsM*, *papA*, *papC*, *papH* and *sfaX*). The ARG group ‘ESBL-other’ includes the genes *bla*_TEM-30_ and *bla*_TEM-235_, the group ‘Beta lactam’ includes genes *bla*_TEM-1_ and *bla*_OXA-1_, and the group pAmpC (plasmid-mediated AmpC) includes the gene *bla*_CMY-138_.

**Fig. 6.**
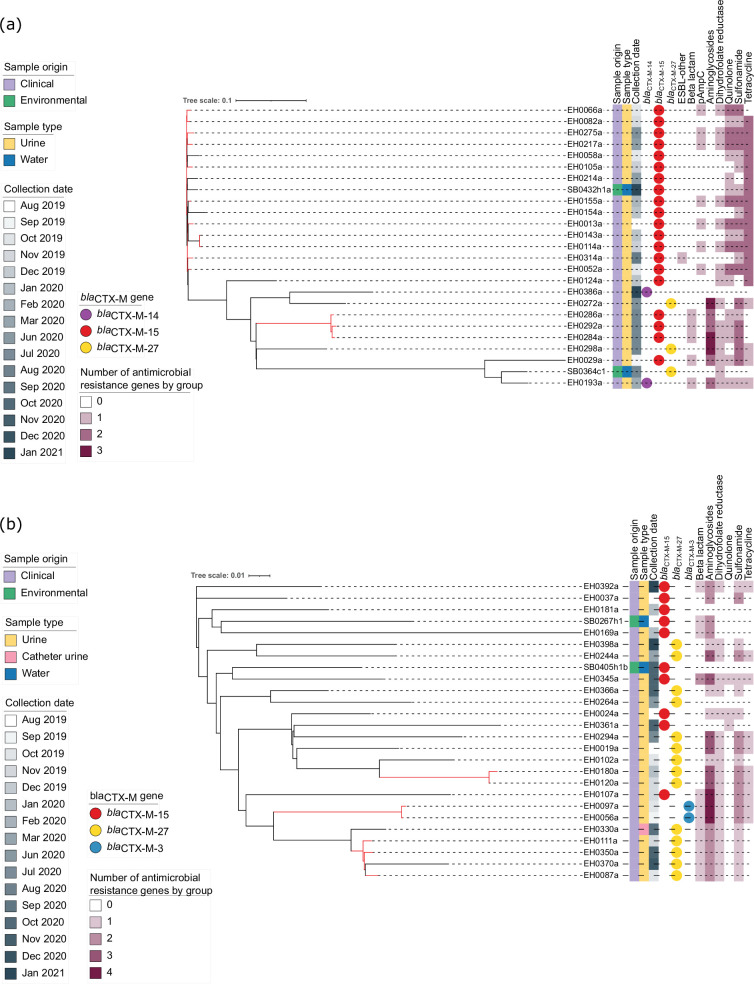
Core SNP phylogeny of ST69 and ST1193 clinical and environmental *E. coli* isolates. Both trees were constructed with FastTree using a maximum likelihood GTR model and visualized in iTOL. The scale bar represents the proportion of nucleotide differences in the core SNP alignment used to calculate the divergence of the isolates. From left to right, the coloured strips represent the sample origin, the sample type and the collection date. The circles represent the *bla*_CTX-M_ gene variant, and the heatmap represents the presence/absence of genes conferring resistance to other groups of antibiotics. (**a**) Maximum likelihood phylogenetic tree of 25 ST69 *E. coli* produced using 630 SNPs with EH0143a used as the reference genome. The ARG group ‘ESBL-other’ includes the gene *bla*_TEM-235_, ‘Beta lactam’ includes the gene *bla*_TEM-1_, and ‘pAmpC’ (plasmid-mediated AmpC) includes the gene *bla*_DHA-1_. (**b**) Maximum likelihood phylogenetic tree of 26 ST1193 isolates produced using 726 SNPs with EH0294a used as the reference genome. The ARG group ‘Beta lactam’ includes the genes *bla*_TEM-1_ and *bla*_OXA-1_.

We explored the clonal spread of both clinical and environmental ESBL-producing *E. coli* within the three dominant STs (ST131, ST69 and ST1193) by performing a core SNP comparison (Figs5  6(Tables S7-S9). There was evidence of clonal spread between humans and the environment for ST131 (Fig. 5) and ST69 (Fig. 6a), but not ST1193 (Fig. 6b). There were four environmental isolates (SB0337h1a, SB0338h1b, SB0432h1a and SB0432h1a) that were closely related (≤10 SNPs) to some of the clinical isolates. All four of these environmental isolates originated from the Manawatū River downstream of the wastewater treatment plant. Three of these four environmental isolates were ST131, and one of these four was ST69. There were fewer than ten SNP differences between the environmental ST131 isolates SB0337h1a and SB0338h1b and ten of the clinical isolates, as well as between SB0405h1a and three of the clinical isolates (Table S7). Two of the clinical strains were received within 14 days of isolation of SB0337h1a and SB0338h1b. There were ten SNP differences between environmental ST69 isolate SB0432h1a and three clinical isolates (EH0052a, EH0082a and EH0155a) (Table S8). Although there was no evidence of clonal spread of ST1193 between humans and the environment, the SNP analysis did suggest that there was human-to-human transmission. Isolates EH0056a and EH0097a were isolated from samples collected on 8 October 2019 and 19 November 2019, respectively, with one SNP difference between them, both containing the *bla*_CTX-M-3_ gene and the same antimicrobial-resistant gene profile (Table S9, Fig. 6b). There were four to five SNP differences between the isolates EH0087a, EH0111a and EH0350a. However, we received isolate EH0111a from MedLab Central 29 days after EH0087a, and EH0350a was received another 11 months later.

In summary, the comparative genomic analysis of human clinical and environmental ESBL-producing *E. coli* isolates identified ST131 as the dominant sequence type, with evidence of clonal spread between humans and the environment for ST131 and ST69. The study revealed specific sequence types unique to either river, effluent or human clinical samples.

### ESBL gene analysis

**Fig. 7.**
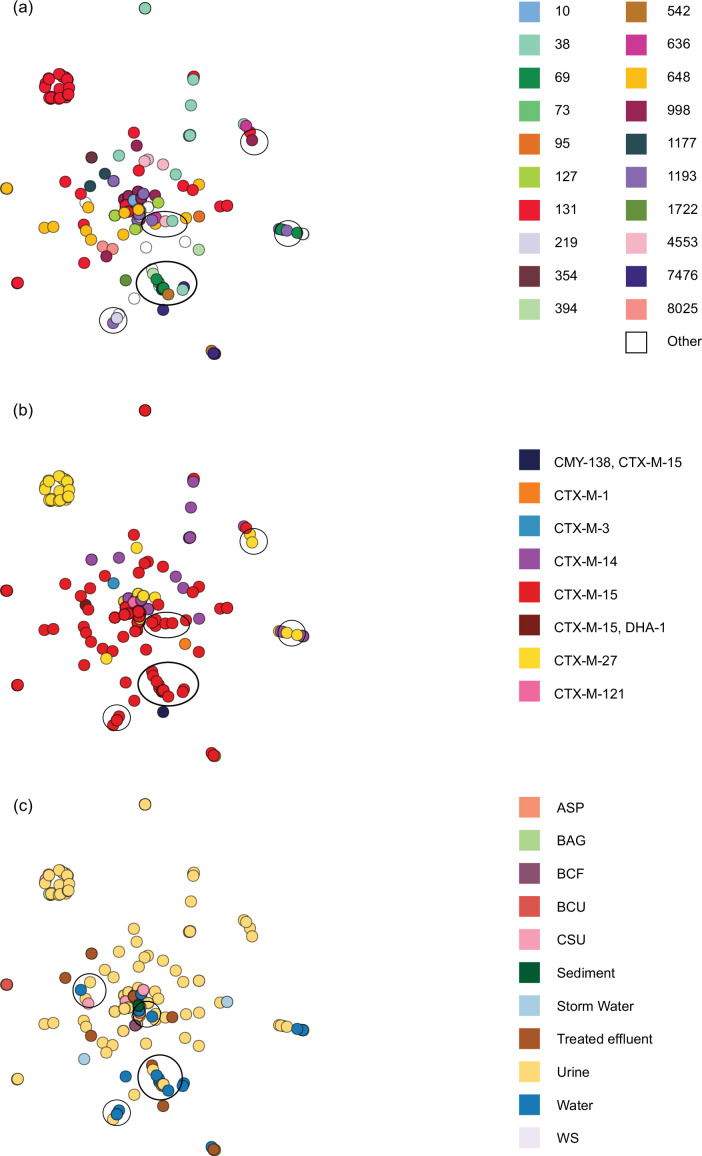
Network analysis of the *bla*_CTX-M_ containing contigs from clinical and environmental *E. coli*. Each circle denotes one contig from one isolate and is coloured by (a) sequence type, (**b**) ESBL type and (**c**) source of isolate. ASP, aspirate; BAG, bag urine; BCF, aspirate fluid (generally blood) in blood culture bottle; BCU, blood culture; CSU, catheter urine; WS, wound swab. For** (a) and (b),** groups of similar contigs containing different STs but the same *bla*_CTX-M_ variant are circled. For **(c)** groups of similar contigs containing contigs from isolates originating from both water and human clinical infections are circled.

**Fig. 8.**
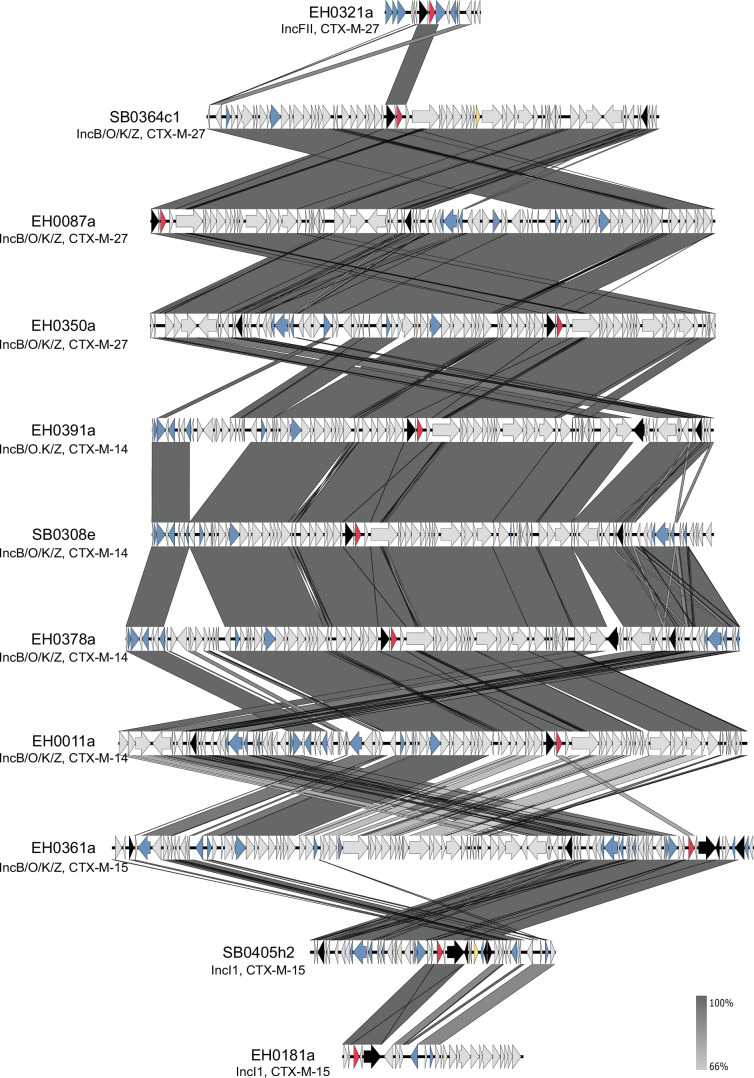
A gene synteny diagram comparing the degree of homology between 11 contigs containing both a plasmid replicon and *bla*_CTX-M_ gene. Transposon and insertion sequences are coloured in black, *bla*_CTX-M_ genes in red, *tra* genes in yellow, hypothetical protein genes in grey and other genes in blue.

To determine whether mobile genetic elements containing the *bla*_CTX-M_ genes were shared within and between sequence types, a network analysis was carried out. We identified several small clusters containing different STs but the same *bla*_CTX-M_ variant (*bla*_CTX-M-27_ and *bla*_CTX-M-15_), suggesting horizontal gene transfer may be occurring between different lineages. There was also some grouping of similar contigs from isolates originating from both water and human infections (Fig. 7c). We examined 11 *bla*_CTX-M_ containing contigs in more detail where a plasmid replicon was also present on the same contig (Fig. 8). These contigs varied in length from 14 381 bp up to 96 109 bp. Eight of these 11 contigs contained an IncB/O/K/Z replicon, 2/11 contained an IncI1 replicon, and 1/11 contained an IncFII replicon.

## Discussion

Our study found that ESBL-producing *E. coli* were frequently isolated from an Aotearoa New Zealand waterway that passes through an urban environment. These bacteria were isolated from at least one sampling site during 12 out of the 13 timepoints. Three of the four sampling sites were associated with urban land use, while one site was associated with agricultural land use. The site with the highest prevalence of ESBL-producing *E. coli* was located downstream of a treated effluent outlet (site F). This is not surprising as it has previously been shown that wastewater treatment is not completely effective at removing antimicrobial-resistant bacteria [66,69], and studies have found a higher prevalence of ESBL-producing *E. coli* and other types of resistant *E. coli* immediately downstream of effluent outlets [32,70,72]. In Aotearoa New Zealand, 44% of wastewater treatment plants release treated effluent into rivers [73]. A higher prevalence of antimicrobial-resistant bacteria and their genes has also been detected during periods associated with higher rainfall [74,76]. One reason for this increase could be effluent overflow, which has been shown to be the dominant contributor to the bacterial community in a river downstream of a wastewater treatment plant after high rainfall events [77]. In our study, ESBL-producing *E. coli* were isolated throughout the year during periods of low and high rainfall.

ESBL-producing *E. coli* were also isolated from sites upstream of the wastewater treatment plant. In Aotearoa New Zealand, it has previously been established that there are antimicrobial-resistant *E. coli* present in freshwater environments [30,35, 78, 79]. Studies suggest that the recreational use of contaminated waters may also be an exposure route for ESBL-producing *E. coli*-associated infections in humans [21,25]. Although the carriage of ESBL-producing *E. coli* has not been found to be higher in recreational water users, previous studies have identified recreational water use as a risk factor for ESBL-producing *E. coli*-associated infections [26,28]. In Aotearoa New Zealand, freshwater bodies are frequently used for recreational use and food harvesting. Watercress is commonly harvested from freshwater and has been found to be a source of MDR *E. coli* [80,81]. The Manawatū River is used for collecting whitebait, trout fishing and swimming. It is estimated that 52% of New Zealanders swim outdoors at least once per year [82]. Although there is little data on the acquisition of ESBL-producing *E. coli*-associated infections from swimming in freshwater, enteropathogenic *E. coli* infections have been associated with swimming in rivers and lakes [83]. Modelling has predicted that a swimmer may swallow between 0.4 and 61 c.f.u. while swimming in a natural water body with high (greater than 1000–5000 c.f.u. l^−1^) concentrations of *E. coli* [21,84].

The most common ESBL-coding gene variant among *E. coli* isolated from waterways differs between studies. Our study found that *bla*_CTX-M-15_ was the dominant gene variant for the treated effluent and water, which concurs with studies carried out in Europe [32,33, 85]. In contrast, a study carried out in Brazil found *bla*_CTX-M-2_ was the dominant gene variant for ESBL-producing *E. coli* isolated from a waterway downstream of a wastewater treatment plant, whereas *bla*_CTX-M-8_ was the main ESBL-coding gene associated with *E. coli* isolated from the wastewater plant [86]. Other *bla*_CTX-M_ variants commonly harboured by ESBL-producing *E. coli* from freshwater include *bla*_CTX-M-1_, *bla*_CTX-M-14_ and *bla*_CTX-M-27_ [87,91].

The most frequently detected ESBL gene types among the clinical isolates were *bla*_CTX-M-27_ 134/307 (43.6%) and *bla*_CTX-M-15,_ 134/307 (43.6%). These findings are similar to a survey recently undertaken in Aotearoa New Zealand, which found 68/158 (43.0%) of human clinical ESBL-producing *E. coli* isolates carried the *bla*_CTX-M-15_ and 64/158 (40.5%) carried the *bla*_CTX-M-27_ gene [6]. A regional survey also found a similar proportion of ESBL-producing *E. coli* carrying *bla*_CTX-M-27_ compared with *bla*_CTX-M-15_, 18/65 (27.7%) and 14/65 (21.5%), respectively [13].

In concordance with other studies [85], other ARGs conferring resistance to a range of other antibiotic classes including aminoglycosides, trimethoprim, sulphonamides, tetracyclines, fluoroquinolones, phenicols and phosphonic antibiotics were present across both the clinical and environmental isolates. Similar plasmid types were also shared across both the clinical and environmental isolates, with IncF being the most common plasmid type. IncF plasmids frequently harbour *bla*_CTX-M_ genes [92]. The close clustering of isolates by the *bla*_CTX-M_ containing contigs across some STs in our study suggests horizontal gene transfer may have occurred between strains. Long-read sequencing of plasmids would be needed to conﬁrm which plasmid Inc types harboured the ESBL-coding genes.

Both treated effluent and water samples from the river contained a diverse range of *E. coli* sequence types, in agreement with previous studies [32,33, 69]. Some of these STs including ST7476 have rarely been isolated from humans. Although the potential impact of human exposure to a diverse range of sequence types through recreational water use is unknown, if exposure is occurring, it is possible that this could provide a route for the dissemination of new high-risk clones.

ST131, which is the main lineage associated with UTIs and blood infections, was the dominant type from the effluent, river samples and clinical isolates. Given the ability of ST131 to adapt and spread [93], the isolation of these strains from freshwater and the potential for an additional exposure route is of concern. In Aotearoa New Zealand, ST131 remains the most prominent ST found in clinical specimens as reported by previous surveys [6,13]. In our study, the majority of ST131 clinical and environmental isolates fell within clade C. In contrast, a recent wastewater surveillance study in Canada found that clade A was the dominant ST131 clade with only 8.6% of strains belonging to clade C [94]. In our study, all the clade C isolates had a fluoroquinolone resistance genotype. Elevated levels of fluoroquinolone resistance are generally associated with mutations in both *gyrA* and *parC* [2,95] and are commonly associated with the C2 clade but are reported to be rare in clade A strains, as was also found in our study [96]. However, a recent study found 72.7% of ST131 clade A strains isolated from wastewater in Canada were resistant to ciprofloxacin [94].

In our study, ST38 and ST648 were also isolated from the three sample types (humans, effluent and the river). ST38 and ST648 are often associated with blood infections and UTIs in humans and have frequently been isolated from waterways and effluent [32,33, 69, 97,99]. A previous study conducted in Sweden also isolated ST38 *E. coli* from water samples downstream of a treated effluent outlet. Other studies that have examined freshwater for the presence of ESBL-producing *E. coli* have found that ST949 and ST10 dominate [33,85]. We did not detect ESBL-producing ST949 *E. coli* in our river samples, and ST10 was isolated from the sediment once during our study. The other dominant ST from our water samples was ST1722. This ST has previously been isolated from humans, effluent, livestock, birds, companion animals and waterways (https://enterobase.warwick.ac.uk/, accessed on 13 February 2024). Interestingly, in our study, ST1722 was only isolated from water. The presence of a diverse range of STs in water may provide a route of exposure for humans to emerging clones.

In this study, we found that ESBL-producing *E. coli* found in an Aotearoa New Zealand waterway were genetically similar to those isolated from human clinical infections, where the difference in the number of SNPs was fewer than or equal to ten SNPs between several human and river isolates. However, our epidemiological data only supported the recent transmission between humans to the local river within one set of isolates, where the dates of isolation of both the human and the river isolates were within 14 days. The close clustering of multiple human clinical isolates also suggests that there was human-to-human transfer or exposure through the same non-human source of ESBL-producing *E. coli* in the community. A limitation of our study is that we only sampled from one geographical area, and within this area, the treated effluent and river collection occurred once a month, whereas our clinical isolates were collected weekly. This may have reduced our ability to detect more ESBL-producing *E. coli* from the treated effluent and river that were genetically similar to the clinical isolates.

Previous studies have also indicated that there is the spread of ESBL-producing *E. coli* from humans to freshwater (or vice versa) [30,32, 85]. Fagerström and Mölling [32] compared ESBL-producing *E. coli* UTI isolates to those sourced from freshwater using a cgMLST approach and found that some isolates had less than ten allele differences, suggesting the sharing of strains between humans and the environment. A study conducted in Germany found that ST949 *E. coli* isolates collected from swimming and bathing sites were closely related to human clinical ST949 isolates from Aotearoa New Zealand and Sweden, although the difference in the number of SNPs or allele changes between isolates was not stated [85]. The most likely source of human-associated ESBL-producing *E. coli* in the environment is through the disposal of treated effluent into our waterways [100].

In conclusion, this study found that ESBL-producing *E. coli* are present in water, sediment, stormwater and treated effluent samples collected along the Manawatū River. It was shown that while treated effluent is an environmental source of antimicrobial-resistant *E. coli*, these resistant bacteria were also present in the Manawatū River upstream of the treated effluent outflow. There was some evidence for the sharing of genetically related ESBL-producing *E. coli* between clinical and environmental sources. The study was limited by the number of isolates collected and sequenced. More frequent sampling would provide a clearer picture of the genetic relatedness between environmental and human ESBL-producing *E. coli* isolates. Our findings emphasize the importance of including an environmental component in antimicrobial resistance surveillance.

## supplementary material

10.1099/mgen.0.001341 Uncited Fig. S1.

10.1099/mgen.0.001341 Uncited Table S1.

## Abbreviations

ARGs: antimicrobial resistance genes
BPW: buffered peptone water
cgMLST: core-genome MLST
CIs: confidence intervals
ECB: *E. coli* broth
ESBL: extended-spectrum beta-lactamase
iTOL: Interactive Tree Of Life
MDR: multidrug-resistant
MLST: multilocus sequence typing
STs: sequence type
