# Supplementary material for: Genomic epidemiology of extended-spectrum beta-lactamase-producing Escherichia coli from humans and a river in Aotearoa New Zealand
Source: Microb Genom. 2025 Jan 10;11(1):001341. doi: 10.1099/mgen.0.001341 (PMC11718517; doi:10.1099/mgen.0.001341)
Supplement: Uncited Fig. S1. [file mgen-11-01341-s001.pdf]

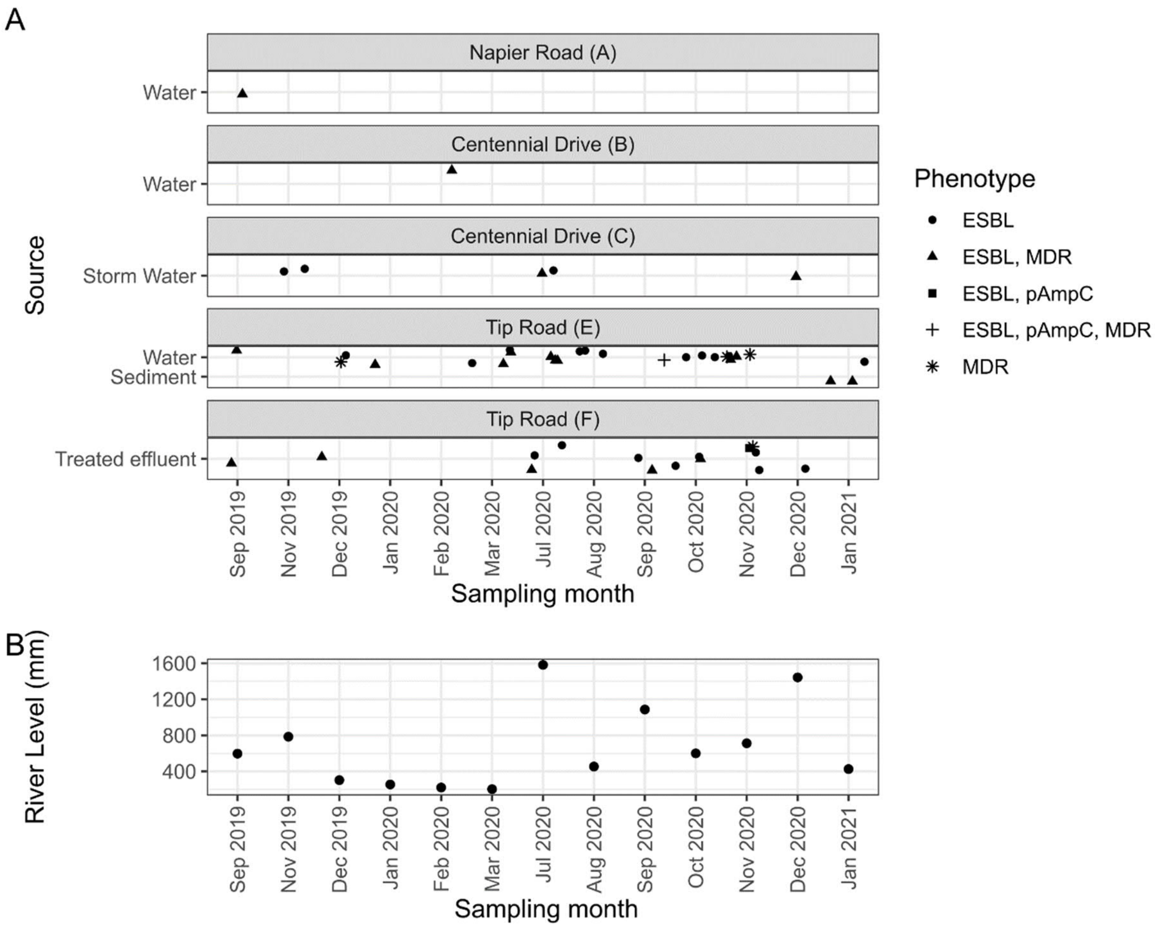


Figure S1. A) Samples positive for ESBL-producing, plasmid-mediated AmpC producing and/or MDR *E. coli* over time. B) River level at the time of sampling.

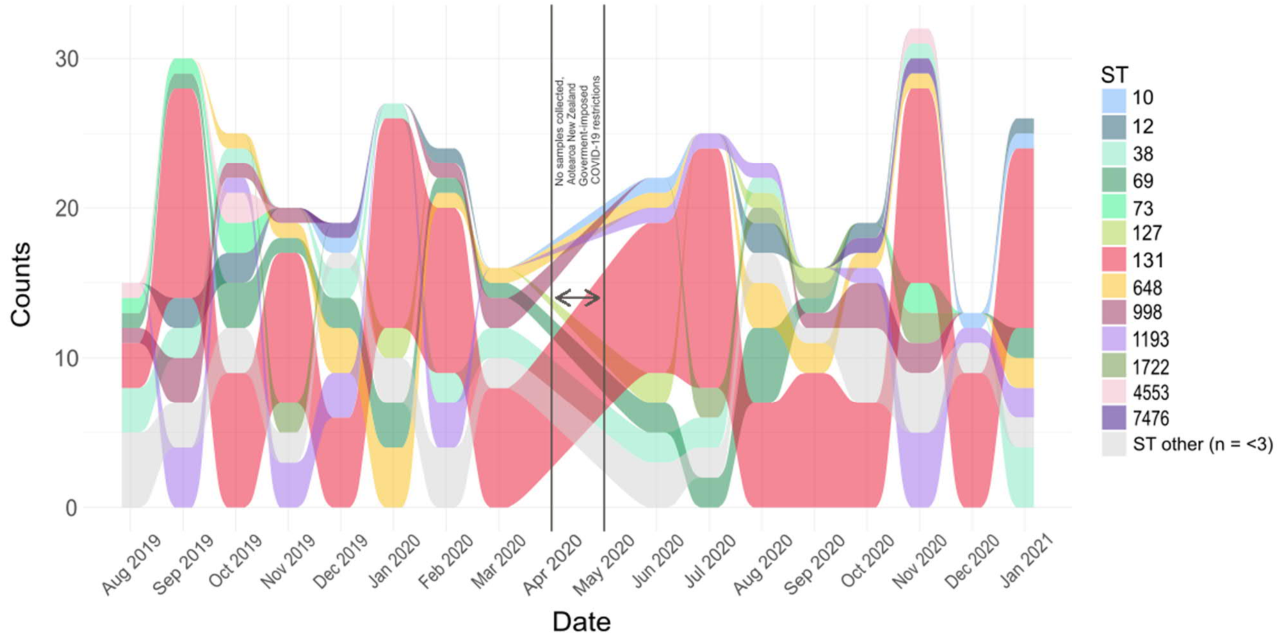


Figure S2. Alluvial plot of the sequence types of ESBL-producing *E. coli* over the sampling time.
